# Clinical Relevance of Initial Hounsfield Unit Values in Predicting Outcomes and Complications of Aneurysmal Subarachnoid Hemorrhage

**DOI:** 10.7759/cureus.92873

**Published:** 2025-09-21

**Authors:** Yushin Takemoto, Yu Hasegawa, Kozo Tashima, Akihito Hashiguchi, Koichi Moroki, Hajime Tokuda, Akitake Mukasa

**Affiliations:** 1 Neurosurgery, Japanese Red Cross Kumamoto Hospital, Kumamoto, JPN; 2 Neurosurgery, Tokuda Neurosurgical Hospital, Kanoya, JPN; 3 Neurosurgery, Kumamoto University Hospital, Kumamoto, JPN; 4 Neurosurgery, Ariakeseijin Hospital, Kumamoto, JPN

**Keywords:** aneurysmal subarachnoid hemorrhage, computed tomography, delayed cerebral ischemia, hounsfield unit value, interpeduncular cistern, prognostic marker, symptomatic vasospasm

## Abstract

Objectives: This study aimed to evaluate whether the initial Hounsfield unit value (IT-HUV), easily obtained from non-contrast CT, serves as a reliable predictor not only for symptomatic vasospasm (SVS) but also for delayed cerebral ischemia (DCI) and overall prognosis in patients with aneurysmal subarachnoid hemorrhage (SAH).

Methodology: A retrospective cohort of 95 patients with aneurysmal SAH who underwent craniotomy clipping and completed rehabilitation between January 2010 and December 2019 was analyzed. Associations between radiological parameters and functional outcomes (modified Rankin Scale (mRS)), as well as DCI and SVS, were assessed using univariate and multivariate analyses.

Results: Of the included patients, 27 (28.4%) had poor outcomes (mRS 4-5), 20 (21.1%) developed DCI, and 19 (20%) experienced SVS. Significant correlations were observed between IT-HUV and mRS (*P *< 0.01), SAH clearance (*P *= 0.02), and Hijdra sum scores (*P *= 0.03); between DCI and IT-HUV (*P *< 0.01), postoperative HUV (*P *= 0.02), and Hijdra sum scores (*P *< 0.01); and between SVS and all variables (SAH clearance: *P* = 0.04; others: *P *< 0.01). IT-HUV was the strongest radiological predictor for mRS, DCI, and SVS, with optimal thresholds around 46.2-46.9.

Conclusions: IT-HUV provides a practical and reliable imaging biomarker for predicting prognosis, DCI, and SVS in patients with aneurysmal SAH. Incorporating IT-HUV into routine assessments may enhance risk stratification and guide individualized treatment strategies.

## Introduction

Early brain injury (EBI) and delayed cerebral ischemia (DCI), as defined by the multidisciplinary consensus of Vergouwen et al. [1], are significant prognostic factors in subarachnoid hemorrhage (SAH), with cerebral vasospasm being a major contributor to DCI [2]. Moreover, previous studies have identified symptomatic vasospasm (SVS), defined by the Japanese consensus [3], age, and severity measured by the World Federation of Neurological Surgeons (WFNS) grade (Drake, 1988) [4] as independent prognostic factors for SAH [5].

Recently, it has been demonstrated that the Hounsfield unit value (HUV) of interpeduncular cistern hematoma can effectively predict SVS, offering a straightforward and practical method for diagnosis [6]. The characteristics of interpeduncular cistern hematoma HUV are believed to reflect the severity of SAH due to their representation of hematoma concentration and density [6-10].

As such, the initial (IT-) HUV could prove valuable in predicting SAH prognosis. However, either surgical intervention, cerebrospinal fluid (CSF) drainage, or both can significantly influence the volume of residual subarachnoid hematoma. Indeed, removing subarachnoid hematoma from the cerebral sulcus during clipping surgery can decrease SVS incidence [11]. Additionally, individual differences exist in SAH clearance (SAH-CL). Furthermore, research indicates that subarachnoid hematoma volume and the rate of hematoma washout per day are the most reliable predictors of SVS [12]. Therefore, we hypothesized that IT-HUV and postoperative (PO-) HUV, along with SAH-CL, could be valuable in predicting outcomes, DCI, and SVS. In this study, we measured the HUV of the interpeduncular cistern at two time points (at admission and one day post-surgery), calculated SAH-CL, and clarified their relationship with outcomes and the incidence of DCI and SVS in patients with aneurysmal SAH.

## Materials and methods

Patient and clinical variables

We conducted a retrospective review of the clinical, radiographic, and laboratory data of patients with SAH who were admitted to our hospital between January 2010 and December 2019. Approval for our study was obtained from the Institutional Review Board of Tokuda Neurosurgical Hospital (In-hospital Clinical Research Registration Number: 0097). This retrospective cohort study of observational data was analyzed according to the STROBE statement. Included patients were those discharged from our hospital after completing in-hospital rehabilitation. Patients’ background data, as well as neurological and radiological findings, were extracted from their charts.

Neurological outcomes were assessed with the modified Rankin scale (mRS), originally described by Rankin [9] and modified by van Swieten et al. [10]. The mRS is Open Access and was applied without restriction. Baseline neurological severity was graded using the WFNS scale, as first proposed by Drake (1988) [4]; this grading system is also Open Access.

In addition, IT- and PO-HUV levels were measured, and SAH-CL was calculated. For HUV measurement, a circular region of interest (ROI) with a diameter of 8 mm was placed in the interpeduncular cistern at the level of the midbrain on non-contrast CT. The mRS score upon completion of in-hospital rehabilitation was set as the primary endpoint, with DCI and SVS as secondary endpoints. DCI was defined per the multidisciplinary consensus definition [1]; this definition is not Open Access, and permission for its use was obtained from the publisher (documentation has been submitted to the support team). SVS was defined according to the 25th Spasm Symposium consensus[3], which is Open Access.

Relationships between patient variables and the primary and secondary endpoints were analyzed. Baseline demographic and clinical data included age, sex, and WFNS grade. All patients underwent surgical clipping within 72 hours. Surgical procedures and perioperative management were performed according to previously described protocols [5,11]. Briefly, surgical clipping was conducted via an appropriate standard craniotomy. Additional treatments such as bypass surgery, suction decompression, anterior clinoidectomy, ventricular or lumbar drainage, and external decompression were performed as necessary. All patients underwent normal water balance management to prevent delayed cerebral ischemia. Rehabilitation commenced as soon as the patient’s condition stabilized post-surgery. Outcomes were assessed at discharge using the mRS, with scores of 4 or 5 defined as unfavorable outcomes.

Radiological variables

The amount of SAH blood on computed tomography (CT) at admission was assessed using the Hijdra sum score, a validated scale assigning scores to 10 cisterns and 4 ventricular regions (0-3 each, total 0-42) [13]. This scoring method is Open Access.

We also measured the maximum size of the ruptured aneurysm on three-dimensional CT angiography (CTA) at admission. SVS was defined as previously reported [3], requiring: (1) the presence of neurological deterioration, such as focal deficit, decreased level of consciousness, and motor paresis; (2) exclusion of other identifiable causes, including intracranial disorders and systemic complications, of neurological worsening; and (3) angiographic evidence of vasospasm (AVS) in the major trunk of the internal carotid artery (ICA), anterior cerebral artery (ACA; A1), middle cerebral artery (MCA; M1), posterior cerebral artery (PCA; P1), and basilar artery (BA). AVS was determined as a diameter reduction of ≤66% on postoperative days 7-10 compared to the diameter at initial admission, as visualized on three-dimensional CTA [3].

DCI was defined as non-surgical infarction or neurological deterioration persisting for more than one hour [1], after exclusion of other causes, following the consensus definition [1]. Cerebral infarction was assessed using diffusion-weighted magnetic resonance imaging between postoperative days 10 and 14 [5,11].

HUV was calculated as previously described [6]. Specifically, HUV was determined using axial CT images acquired on admission after onset (IT-HUV) and on postoperative day 1 (PO-HUV). CT scans were conducted using an Aquilion scanner with a 0.5 mm thickness and 80 slices (Canon, Tokyo, Japan). A region of interest (ROI) was defined in the interpeduncular cistern, including the basal cistern, on a selected slice. To minimize partial volume effects and assess only hematoma density, a relatively small circle diameter of 8 mm was employed for the ROI (Appendix A) [6]. The average HUV for the ROI was evaluated by an author who was blinded (YT) to other clinical information, following instructions provided by another author (YH).

SAH-CL was calculated using the following formula:

\[ \text{SAH-CL (%)} = \frac{\text{IT-HUV} - \text{PO-HUV}}{\text{IT-HUV}} \times 100 \]

Statistical analysis

All data were analyzed using GraphPad Prism version 10 for Mac (GraphPad Software, San Diego, CA) and Mac Multivariate Analysis ver.3 (ESUMI, Tokyo, Japan). To conduct dichotomous analysis, we established thresholds based on our previous report [5,6] and other pertinent literature, as follows: age (≥65 or <65 years), WFNS (I-III or IV-V), Hijdra sum score (0-19 or 20-30), aneurysm size (≥7 mm or <7 mm), and mRS (0-3 or 4-5) [5,9,10,13-15]. The chi-square test was used for categorical variables and the Mann-Whitney U test for continuous variables in the univariate analysis. In multivariate analysis, multiple regression analysis was performed to assess the combined effects of the parameters on the primary and secondary endpoints. Receiver operating characteristic (ROC) curves were generated to determine the thresholds of relevant radiological parameters for the primary and secondary endpoints. *P*-values <0.05 were considered statistically significant.

## Results

Clinical characteristics

**Figure 1:**
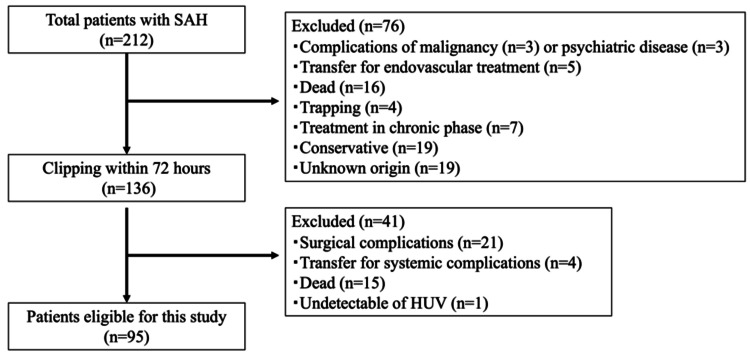
Flow diagram of patient inclusion and exclusion criteria. SAH, subarachnoid hemorrhage; HUV, Hounsfield unit value

A total of 212 patients with SAH were admitted to our hospital. Exclusion criteria included patients with malignancy (*n *= 3, 1.4%) and psychiatric disease (*n* = 3, 1.4%) that could affect the course of treatment and outcome; those who did not undergo surgical clipping within 72 hours after SAH due to preoperative death (*n* = 16, 7.5%), observation only for fatal SAH (*n* = 19, 9.0%), unknown origin (*n* = 19, 9.0%), surgical treatment in the chronic phase (*n *= 7, 3.3%), or surgical trapping (*n *= 4, 1.9%); those who underwent endovascular treatment at other hospitals since no endovascular specialist was available at our institution (*n *= 5, 2.4%); postoperative death (*n *= 41, 19.3%); systemic complications such as heart failure (*n *= 2, 0.9%), pneumonia (*n* = 1, 0.5%), or ileus (*n* = 1, 0.5%); surgical complications, including ischemia (*n* = 16, 7.5%) and hemorrhage (*n *= 5, 2.4%); or undetectable HUV levels (*n* = 1, 0.5%). Finally, a total of 95 patients (44.8%) with aneurysmal SAH who underwent surgical clipping within 72 hours and received subsequent in-hospital rehabilitation were included in this study. A flow diagram of patient selection is presented in Figure 1.

The mean age was 66.2 years, and 72 patients (75.8%) were female. WFNS grades I-III were observed in 71 patients (74.7%). Hijdra sum scores of 0-19 were found in 62 patients (65.3%). Ruptured aneurysm locations were as follows: ACA, 38 patients (40.0%); ICA, 33 (34.7%); MCA, 20 (21.2%); and vertebral artery-basilar artery, 4 (4.2%). The mean size of the ruptured aneurysms was 5.7 mm (range, 2.0-13.1 mm). SVS occurred in 19 patients (20.0%), and DCI in 20 patients (21.1%). mRS scores of 0-3 were reported in 68 patients (71.6%), while scores of 4-5 were observed in 27 (28.4%). These baseline demographic and clinical characteristics are summarized in Table 1.

**Table 1:** Patients’ background characteristics. AN, aneurysm; DCI, delayed cerebral ischemia; IT-HUV, initial Hounsfield unit value; mRS, modified Rankin scale; PO-HUV, postoperative Hounsfield unit value; SAH-CL, subarachnoid hemorrhage; SVS, symptomatic vasospasm; WFNS, World Federation of Neurological Surgeons; ACA, anterior cerebral artery; ICA, internal carotid artery; MCA, middle cerebral artery; VA-BA, vertebral artery-basilar artery

Characteristics	Category	Value (Mean (range)/*n* (%))
Age (years)	Mean (range)	66.2 (29-96)
	>=65	50 (52.6)
Sex	Female	72 (75.8)
	Male	23 (24.2)
WFNS grade	Ⅰ-Ⅲ	71 (74.7)
	Ⅳ, Ⅴ	24 (25.3)
Hijdra sum score	0-19	62 (65.3)
	20-30	33 (34.7)
Location	ACA	38 (40.0)
	ICA	33 (34.7)
	MCA	20 (21.1)
	VA-BA	4 (4.2)
AN size (mm)	Mean (range)	5.7 (2.0-13.1)
SVS	*n* (%)	19 (20.0)
DCI	*n* (%)	20 (21.1)
mRS	0-3	68 (61.6)
	4,5	27 (28.4)
IT-HUV	Mean (range)	37.3 (10.6-63.9)
PO-HUV	Mean (range)	32.7 (10.3-60.7)
SAH-CL %)	Mean (range)	0.1 (-1.1 to 0.5)

Univariate analysis of factors related to mRS, DCI, and SVS

In the univariate analysis, age, WFNS grade, Hijdra sum score, aneurysm size, IT-HUV, and SAH-CL were correlated with unfavorable outcomes on the mRS (Table 2).

**Table 2:** Univariate analysis of patients’ characteristics correlated to mRS. Test statistic = χ² for categorical variables, *U* for continuous variables. AN, aneurysm; IT-HUV, initial Hounsfield unit value; mRS, modified Rankin scale; PO-HUV, postoperative Hounsfield unit value; SAH-CL, subarachnoid haemorrhage; WFNS, World Federation of Neurological Surgeons

Variables	Category	mRS 0-3, *n *(%)/Mean (range)	mRS 4-5, *n *(%)/Mean (range)	Test statistic	*P*-value
Age (years)	65	28 (41.2)	22 (81.5)	χ² (1) = 12.59	<0.01
Sex	Female	50 (73.5)	22 (81.5)	χ² (1) = 0.666	0.71
WFNS grade	Ⅳ, Ⅴ	7 (10.3)	17 (63.0)	χ² (1) = 28.39	<0.01
Hijdra sum score	20-30	19 (27.9)	14 (51.9)	χ² (1) = 4.874	0.03
AN size (mm)	7	11 (16.2)	12 (44.4)	χ² (1) = 8.416	<0.01
IT-HUV	Mean (range)	34.5 (10.6-56.0)	44.1 (17.7-63.9)	*U* = 551	<0.01
PO-HUV	Mean (range)	31.3 (11.4-60.7)	36.1 (10.3-60.5)	*U* = 711	0.09
SAH-CL	Mean (range)	0.04 (-1.08 to 0.48)	0.18 (-0.46 to 0.53)	*U* = 598	<0.01

Regarding the secondary endpoints, WFNS grade, Hijdra sum score, IT-HUV, and PO-HUV were correlated with the incidence of DCI (Table 3).

**Table 3:** Univariate analysis of patients’ characteristics correlated to DCI. Test statistic = χ² for categorical variables, U for continuous variables. AN, aneurysm; DCI, delayed cerebral ischemia; IT-HUV, initial Hounsfield unit value; PO-HUV, postoperative Hounsfield unit value; SAH-CL, subarachnoid hemorrhage; WFNS, World Federation of Neurological Surgeons

Variables	Category	DCI (-), *n* (%)/Mean (range)	DCI (+), *n* (%)/Mean (range)	Test statistic	*P*-value
Age (years)	65	36 (48.0)	14 (70.0)	χ² (1) = 3.065	0.08
Sex	Female	56 (74.7)	16 (80.0)	χ² (1) = 0.2448	0.62
WFNS grade	Ⅳ, Ⅴ	13 (17.3)	11 (55.0)	χ² (1) = 11.86	<0.01
Hijdra sum score	20-30	21 (28.9)	12 (60.0)	χ² (1) = 7.132	<0.01
AN size (mm)	7	15 (20.0)	8 (40.0)	χ² (1) = 3.442	0.06
IT-HUV	Mean (range)	34.8 (10.6-63.9)	46.4 (18.2-63.2)	*U* = 393	<0.01
PO-HUV	Mean (range)	31.2 (11.4-60.7)	38.2 (10.3-58)	*U* = 499	0.02
SAH-CL	Mean (range)	0.05 (-1.08 to 0.53)	0.18 (-0.11 to 0.43)	*U* = 536	0.05

In contrast, age, WFNS grade, Hijdra sum score, aneurysm size, IT-HUV, PO-HUV, and SAH-CL were correlated with the incidence of SVS (Table 4).

**Table 4:** Univariate analysis of patients’ characteristics correlated to SVS. Test statistic = χ² for categorical variables, U for continuous variables. AN, aneurysm; IT-HUV, initial Hounsfield unit value; PO-HUV, postoperative Hounsfield unit value; SAH-CL, subarachnoid hemorrhage clearance; SVS, symptomatic vasospasm; WFNS, World Federation of Neurological Surgeons

Variables	Category	SVS (-), *n* (%)/Mean (range)	SVS (+), *n* (%)/Mean (range)	Test statistic	*P*-value
Age (years)	65	36 (47.4)	14 (73.7)	χ² (1) = 4.222	0.04
Sex	Female	56 (73.7)	16 (84.2)	χ² (1) = 0.9179	0.34
WFNS grade	Ⅳ, Ⅴ	13 (17.1)	11 (57.9)	χ² (1) = 13.39	<0.01
Hijdra sum score	20-30	21 (27.6)	12 (63.2)	χ² (1) = 8.462	<0.01
AN size (mm)	7	15 (19.7)	8 (42.1)	χ² (1) = 4.145	0.04
IT-HUV	Mean (range)	34.9 (10.6-63.9)	46.8 (18.2-63.2)	*U* = 363	<0.01
PO-HUV	Mean (range)	31.3 (11.4-60.7)	38.3 (10.3-58)	*U* = 480	<0.01
SAH-CL	Mean (range)	0.05 (-1.08 to 0.53)	0.18 (-0.11 to 0.43)	*U* = 506	0.04

Multivariate analysis of prognostic factors for primary and secondary endpoints

Before conducting the logistic regression analysis, we determined the appropriate number of variables as follows. First, we selected variables showing significant differences in the univariate analysis and those identified in a previous study [5]. Among these, we considered parameters that exhibited significant correlations with prognosis, such as age, WFNS grade, and SVS. Next, we aimed to clarify the relationship between radiological parameters, including HUV, and the primary and secondary endpoints for SAH. The Hijdra sum score, which differs from the Fisher grading scale [16,17], has recently been reported to be associated with prognosis in SAH and SVS [18-20]. Therefore, four radiological variables (IT-HUV, PO-HUV, SAH-CL, and Hijdra sum score) were included in the analysis. To ensure the reliability of the statistical analysis, we selected appropriate candidates for multivariate analysis based on the results of the primary endpoint. Ultimately, IT-HUV, PO-HUV, SAH-CL, Hijdra sum score, age, WFNS grade, and SVS were entered into the multiple regression analysis. Independent unfavorable prognostic factors for mRS were WFNS grade (*P *< 0.001), SVS (*P* < 0.001), and age (*P *< 0.001). Independent prognostic factors for DCI and SVS were SVS (*P *< 0.001) and WFNS grade (*P *= 0.010). None of the radiological variables were independent prognostic factors for the primary and secondary endpoints in the multivariate analysis (Appendices B-D).

ROC analysis of the optimal thresholds for predicting mRS, DCI, and SVS

**Figure 2:**
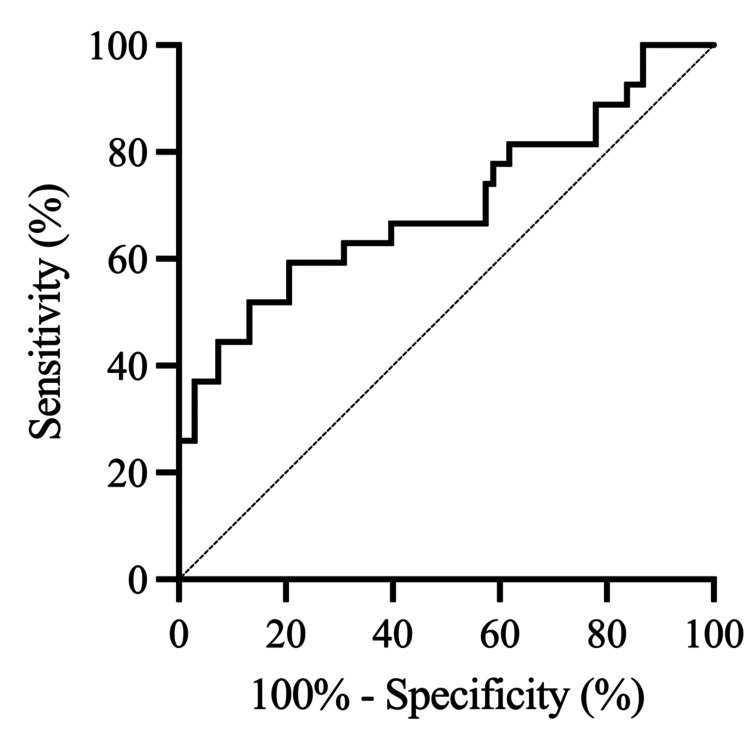
Receiver operating characteristic (ROC) curve of IT-HUV for predicting poor outcomes after in-hospital rehabilitation for SAH. The ROC curve shows that IT-HUV is the most valid radiological prognostic factor, with an optimal threshold of 46.9, a sensitivity of 59.3% and a specificity of 79.4% for predicting poor outcomes on the mRS. SAH, subarachnoid hemorrhage; IT-HUV, initial Hounsfield unit value; mRS, modified Rankin scale

**Figure 3:**
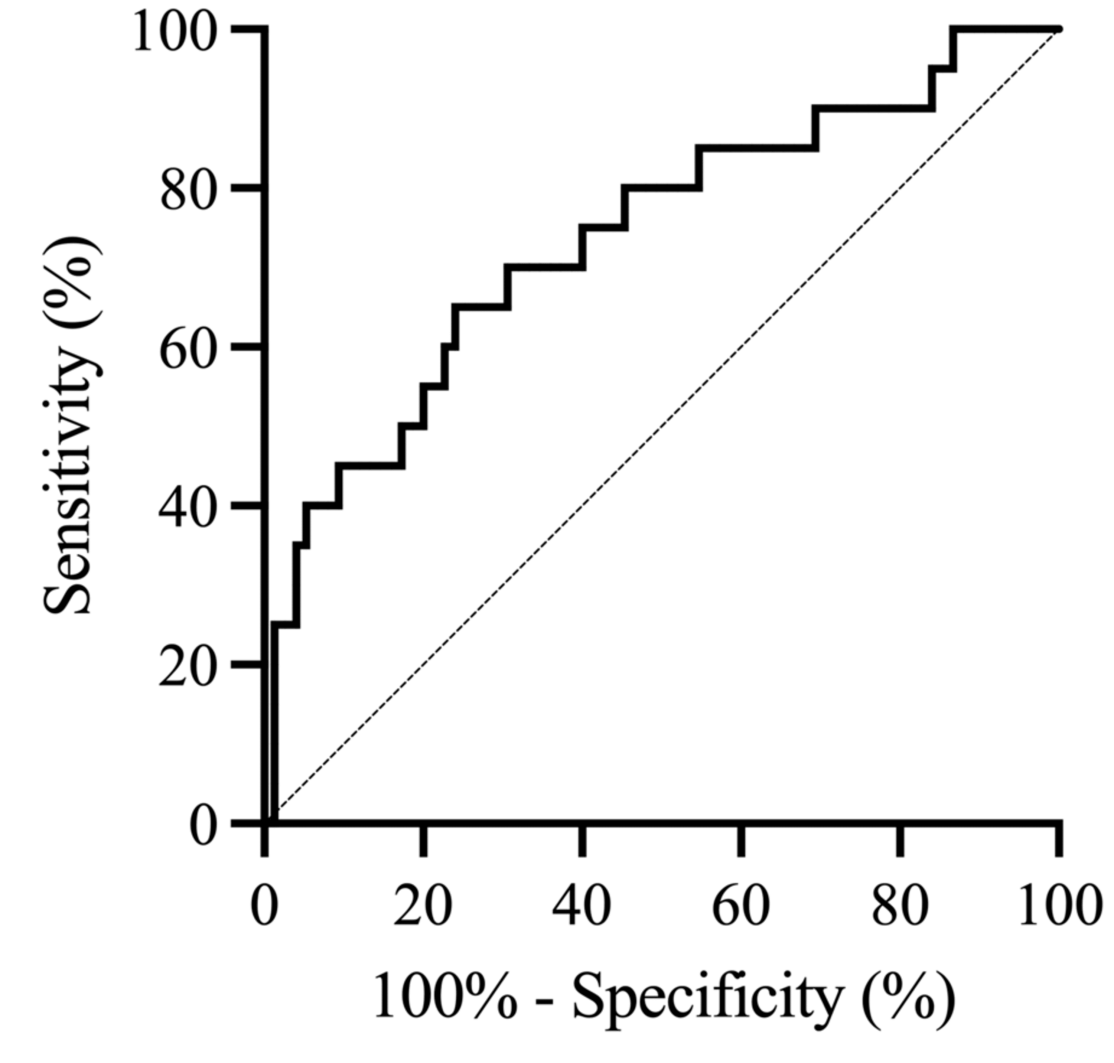
Receiver operating characteristic (ROC) curve of IT-HUV for predicting DCI after in-hospital rehabilitation for SAH. The ROC curve shows that IT-HUV has an optimal threshold of 46.2, with a sensitivity of 65.0% and a specificity of 76.0% for predicting DCI. ROC, receiver operating characteristic; SAH, subarachnoid hemorrhage; IT-HUV, initial Hounsfield unit value; DCI, delayed cerebral ischemia

**Figure 4:**
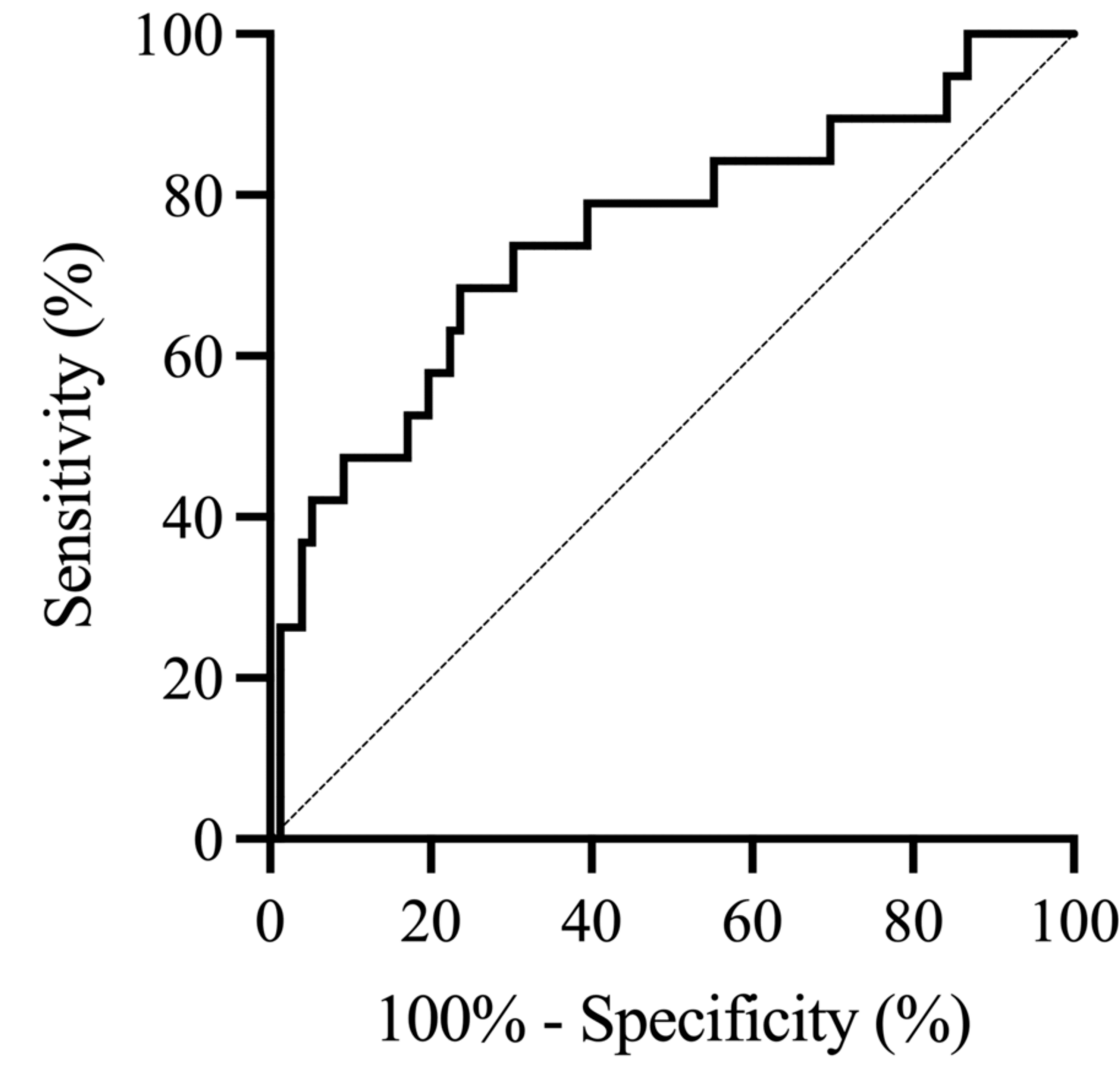
Receiver operating characteristic (ROC) curve of IT-HUV for predicting SVS after in-hospital rehabilitation for SAH. The ROC curve shows that IT-HUV has an optimal threshold of 46.2, with a sensitivity of 68.4% and a specificity of 76.3% for predicting SVS. ROC, receiver operating characteristic; SAH, subarachnoid hemorrhage; IT-HUV, initial Hounsfield unit value; SVS, symptomatic vasospasm

ROC analysis was performed to determine the optimal thresholds for predicting the primary and secondary endpoints following in-hospital rehabilitation for SAH. Among the radiological variables studied (IT-HUV, PO-HUV, SAH-CL, and Hijdra sum score), IT-HUV emerged as the most reliable prognostic factor for poor outcomes on the mRS, DCI, and SVS. The optimal thresholds for IT-HUV were 46.9 for predicting poor outcomes on the mRS (area under the ROC curve (AUC): 0.70; sensitivity: 59.3%; specificity: 79.4%) (Figure 2, Table 5), 46.2 for predicting DCI (AUC: 0.74; sensitivity: 65.0%; specificity: 76.0%) (Figure 3, Table 6), and 46.2 for predicting SVS (AUC: 0.75; sensitivity: 68.4%; specificity: 76.3%) (Figure 4, Table 7).

**Table 5:** ROC analysis for predicting poor outcomes (mRS 4-5). Receiver operating characteristic (ROC) analysis of radiological variables for predicting poor outcomes (mRS 4-5) after in-hospital rehabilitation for SAH. SAH-CL, subarachnoid hemorrhage clearance; IT-HUV, initial Hounsfield unit value; AUC, area under the ROC curve; CI, confidence interval

Variable	AUC	95% CI	*P*-value
IT-HUV	0.70	0.57-0.83	0.0025
SAH-CL	0.67	0.56-0.79	0.0083
Hijdra sum score	0.68	0.56-0.81	0.0054

**Table 6:** ROC analysis for predicting DCI. Receiver operating characteristic (ROC) analysis of radiological variables for predicting delayed cerebral ischemia (DCI) after in-hospital rehabilitation for SAH. SAH, subarachnoid hemorrhage; AUC, area under the ROC curve; CI, confidence interval

Variable	AUC	95% CI	*P*-value
IT-HUV	0.74	0.61-0.87	0.0011
PO-HUV	0.67	0.53-0.80	0.0219
Hijdra sum score	0.71	0.59-0.83	0.0039

**Table 7:** ROC analysis for predicting SVS. Receiver operating characteristic (ROC) analysis of radiological variables for predicting symptomatic vasospasm (SVS) after in-hospital rehabilitation for SAH. AUC, area under the ROC curve; CI, confidence interval

Variable	AUC	95% CI	*P*-value
IT-HUV	0.75	0.61-0.88	0.0008
PO-HUV	0.67	0.53-0.81	0.0243
SAH-CL	0.65	0.53-0.77	0.0445
Hijdra sum score	0.72	0.59-0.84	0.0038

## Discussion

Our results identified IT-HUV as a useful prognostic factor for aneurysmal SAH, with a threshold of 46.9. IT-HUV was also more reliable than PO-HUV and SAH-CL in predicting DCI and SVS, with a threshold of 46.2 for both. These findings suggest that prognosis and the incidence of DCI and SVS can be predicted using non-contrast CT. However, in the multivariate analysis, IT-HUV was not an independent predictor of prognosis; therefore, caution should be exercised when evaluating prognosis, DCI, or SVS based on HUV alone. Nevertheless, IT-HUV correlated well with mRS, DCI, and SVS in the ROC analysis, suggesting its potential utility for predicting complications and outcomes due to its ease of measurement.

Previous studies have reported associations between hematoma volume and SVS or DCI [21-27]. Suzuki et al. reported that HUV >60 was associated with cerebral infarction in patients with SAH [27]. Kanazawa et al. reported that HUV ≥49.95 was correlated with DCI [24], and Ishihara et al. demonstrated that HUV ≥50 was significantly associated with SVS [6]. Hu et al. found that a combination of HUV and white blood cell (WBC) count provided the best predictive performance for DCI, with an HUV threshold of 58.61, while HUV alone also emerged as an independent prognostic factor [23]. This supports the feasibility of predicting DCI using only non-contrast CT.

Park and Kang reported that interpeduncular cistern HUV and Sylvian fissure HUV were associated with SVS in patients undergoing endovascular treatment for mild SAH, with an interpeduncular cistern HUV threshold of 44.875 (sensitivity: 61.5%; specificity: 65.9%) [26]. This threshold is similar to our findings in patients treated with craniotomy clipping, suggesting no substantial difference in the predictive role of HUV between endovascular and surgical cohorts. They also noted that Sylvian fissure HUV was associated with age and outcome in multivariate analysis [26].

In the present study, both IT-HUV and PO-HUV were associated with SVS and DCI. Other reports have shown that subarachnoid hematoma volume and the daily clearance rate of hematoma are strong predictors of SVS [12] and that surgical removal of hematoma reduces SVS incidence [11]. In our study, SAH-CL was associated with SVS but not with DCI (Tables 3 and 4). A possible explanation is that surgical or natural washout occurring several hours after onset did not sufficiently influence DCI, since the pathophysiology of DCI involves EBI as well as SVS. Patients with denser hematomas at baseline may have undergone more extensive removal or faster clearance, which could explain the observed associations.

The Hijdra sum score was also associated with both the primary and secondary endpoints, but ROC analysis indicated that its predictive performance was inferior to IT-HUV. This may be due to two reasons. First, the Hijdra sum score estimates hematoma volume as it appears within the cerebrospinal fluid spaces, making it less precise. Second, unlike the Hijdra sum score, IT-HUV may reflect the concentration and density of the hematoma, which are linked to the intensity of the initial rupture [6,7]. We previously reported that WFNS grade, along with age and SVS, is a predictor of functional outcome [5]. The present findings confirm that IT-HUV is related to WFNS grade and SVS and tends to be related to age (Appendix E). As IT-HUV reflects not only hematoma volume but also hemorrhage intensity at rupture, it may serve as an indicator of EBI due to transient global ischemia associated with sudden intracranial hypertension at onset.

This study has several limitations. First, HUV may not accurately represent hematoma severity in patients with localized clots, such as those confined to the Sylvian fissure or posterior fossa. Careful patient selection may allow HUV to function as an independent predictor of outcome and complications. Nevertheless, this simple CT-based measure may be widely applicable in clinical practice. The interpeduncular cistern, being closest to the Circle of Willis, is considered a feasible site as it best reflects the impact of SAH on the major cerebral arteries [6,23,24,26,27]. 

Second, we did not evaluate MRI or DSA findings as potential predictors of mRS, DCI, or SVS. These modalities are not routinely available for all SAH patients, so they were excluded. Finally, patients who did not complete the rehabilitation program were not included, which may have introduced bias.

Despite these limitations, this study is the first to demonstrate that IT-HUV at admission, easily measured on non-contrast CT, the most common diagnostic tool for SAH, can serve as a simple and clinically useful indicator of prognosis, DCI, and SVS.

## Conclusions

In this study, we evaluated the clinical relevance of a simple radiological parameter in patients with aneurysmal SAH. Our findings demonstrate that the IT-HUV measured on initial non-contrast CT is associated with key outcomes, including DCI, SVS, and functional prognosis. This association may reflect the pathophysiological link between EBI and subsequent complications. Although IT-HUV was not identified as an independent predictor in multivariate analysis, it may serve as a practical and supportive marker when interpreted in conjunction with established prognostic factors such as age, WFNS grade, and SVS.

## Figures and Tables

**Figure 5:**
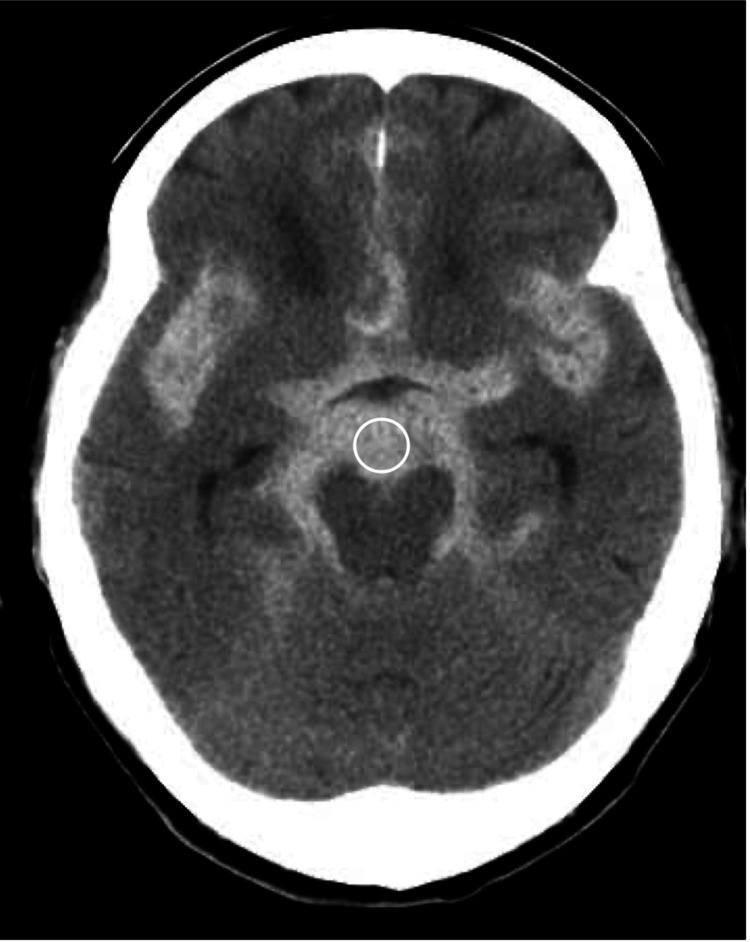
Region of interest (ROI) placement for HUV measurement in the interpeduncular cistern. Using non-contrast CT, the region of interest (ROI) was placed on the interpeduncular cistern at a level containing the midbrain. The white circle indicates an example of HUV measurement. The ROI was defined as a circle of 8 mm diameter. ROI, region of interest; CT, computed tomography; HUV, Hounsfield unit value.

**Figure 6:**
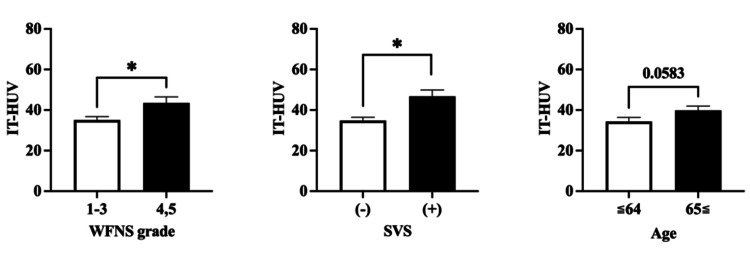
Initial Hounsfield Unit Value (IT-HUV) according to WFNS grade, presence of SVS, and age. The IT-HUV in patients with WFNS grades 4 and 5 was significantly higher than in those with WFNS grades 1–3 (p < 0.05). Similarly, the IT-HUV in the SVS-positive group was significantly higher than in the SVS-negative group (p < 0.05). By contrast, the IT-HUV in patients aged 65 years or older tended to be higher than in those younger than 65 years (p = 0.0583). IT-initial; HUV, Hounsfield unit value; WFNS, World Federation of Neurological Surgeons; SVS, symptomatic vasospasm.

## Appendices

Appendix A

Appendix B 

**Table 8:** Multivariate analysis of independent prognostic factors for mRS. IT-HUV, initial Hounsfield unit value; mRS, modified Rankin scale; PO-HUV, post-operative Hounsfield unit value; SAH-CL, subarachnoid haemorrhage; SVS, symptomatic vasospasm; WFNS, World Federation of Neurological Surgeons.

Variables	Regression coefficient	Standard regression coefficient	F value	P value	95% confidence interval	
					Lower	Upper
IT-HUV	-0.037	-0.518	1.553	0.216	-0.095	0.022
PO-HUV	0.017	0.206	0.289	0.592	-0.045	0.079
SAH-CL	0.556	0.052	0.346	0.558	-1.321	2.433
Hijdra sum score	0.013	0.076	0.303	0.583	-0.034	0.060
WFNS	0.536	0.482	31.367	<0.001	0.346	0.726
SVS	1.129	0.182	13.301	<0.001	0.514	1.743
Age	0.024	0.575	24.408	<0.001	0.014	0.034

Appendix C

**Table 9:** Multivariate analysis of independent prognostic factors for DCI. DCI, delayed cerebral ischemia; IT-HUV, initial Hounsfield unit value; PO-HUV, post-operative Hounsfield unit value; SAH-CL, subarachnoid haemorrhage; SVS, symptomatic vasospasm; WFNS, World Federation of Neurological Surgeons.

Variables	Regression coefficient	Standard regression coefficient	F value	P value	95% confidence interval	
					Lower	Upper
IT-HUV	-0.001	-0.075	0.058	0.811	-0.008	0.007
PO-HUV	-0.001	-0.037	0.017	0.897	-0.008	0.007
SAH-CL	-0.032	-0.018	0.073	0.788	-0.269	0.205
Hijdra sum score	0.001	0.046	0.204	0.652	-0.005	0.007
WFNS	0.005	-0.028	0.192	0.663	-0.029	0.019
SVS	0.990	0.946	641.0	<0.001	0.912	1.067
Age	0.001	0.144	2.720	0.102	0.000	0.002

Appendix D 

**Table 10:** Multivariate analysis of independent prognostic factors for SVS. IT-HUV, initial Hounsfield unit value; PO-HUV, post-operative Hounsfield unit value; SAH-CL, subarachnoid haemorrhage; SVS, symptomatic vasospasm; WFNS, World Federation of Neurological Surgeons.

Variables	Regression coefficient	Standard regression coefficient	F value	P value	95% confidence interval	
					Lower	Upper
IT-HUV	0.007	0.631	0.470	0.495	-0.013	0.028
PO-HUV	-0.003	-0.232	0.076	0.783	-0.024	0.018
SAH-CL	0.003	0.002	0.000	0.992	-0.632	0.639
Hijdra sum score	-0.001	-0.026	0.007	0.935	-0.017	0.016
WFNS	0.083	0.469	6.886	0.010	0.020	0.145
Age	0.007	0.631	0.470	0.495	-0.013	0.028

Appendix E
